# Altered astrocytic autophagy as a potential link between compromised vasculature and demyelination

**DOI:** 10.1002/alz70855_098988

**Published:** 2025-12-23

**Authors:** Gerald Wai‐Yeung Cheng, Hexiao Ding, Kai‐Hei Tse, Jung Sun Yoo

**Affiliations:** ^1^ Hong Kong Polytechnic University, Kowloon, Kowloon, Hong Kong; ^2^ University of Technology Sydney, Sydney, NSW, Australia

## Abstract

**Background:**

Astrocytes are integral in maintaining tight junctions of the blood‐brain barrier while supplying cholesterol to oligodendrocytes for myelination. It is well‐recognized that vascular infarctions and white matter loss are signs of Alzheimer's disease (AD). Previously, we have shown that lipid‐carrier apolipoprotein E isoform ε4 (ApoE4) was positively correlated with demyelination. Furthermore, oligodendrocyte loss and demyelination were shown to accelerate amyloid plaque deposition. However, the ApoE4 allele is not prevalent, only 25% are heterozygous and 3% homozygous, yet 95% of AD are sporadic. Thus, a novel AD pathway is necessary.

Atherosclerosis is a condition in which plaques form in the inner linings of the vasculature, causing increased arterial pressure and risk of cerebral infarctions. The deposition of plaques prevents sufficient laminar shear stress, suppressing autophagy of the endothelial cells and, correspondingly, astrocytic autophagy. Consequently, the functions of astrocytes are hindered, one of which is its production and transport of cholesterol. Here, we hypothesize that atherosclerotic plaques would inhibit proper endothelial and astrocytic autophagy, resulting in improper cholesterol synthesis and transportation to oligodendrocytes and leading to demyelination.

**Method:**

In this study, we employed 7‐month‐old C57BL/6 controls (*n* = 6) with age‐matched ApoE knockout mice (ApoeKO) (*n* = 6) to assess the impact of atherosclerosis on myelination. Quantitative analysis of white matter integrity was performed with T2‐weighted and magnetic resonance spectroscopy sequences from Bruker BioSpec 70/20 USR7T. Y‐maze and Promethion Metabolic Cage were used to investigate changes in short‐term spatial memory and metabolic parameters, respectively. A battery of histochemistry was performed for quantitative assessments of atherosclerotic plaques, astrocytic and endothelial autophagy, myelination, and associated cell types.

**Result:**

Preliminary results show average respiration quotient of 0.94 for ApoeKO and 0.75 for control. We identified significant increase of Oil Red O lipid deposition in grey matter vasculature in ApoeKO indicative of atherosclerotic plaques. Immunohistochemistry shows a reduction in endothelial cell autophagy marker, TRIM47, in ApoeKO gross grey matter with no changes in astrocyte numbers.

**Conclusion:**

On‐going confirmation of the impact of endothelial and astrocytic autophagy with myelination will elucidate underlying mechanisms of sporadic AD progression. Understanding the role of autophagy of astrocytes may highlight additional factors affecting neurodegenerative disorders.

